# Supplementary material for: Ocean acidification conditions increase resilience of marine diatoms
Source: Nat Commun. 2018 Jun 13;9:2328. doi: 10.1038/s41467-018-04742-3 (PMC5997998; doi:10.1038/s41467-018-04742-3)
Supplement: Supplementary file 1 — Supplementary Information [file 41467_2018_4742_MOESM1_ESM.pdf]

SUPPLEMENTARY INFORMATION

Ocean acidification conditions increase resilience of marine diatoms

Valenzuela et al.

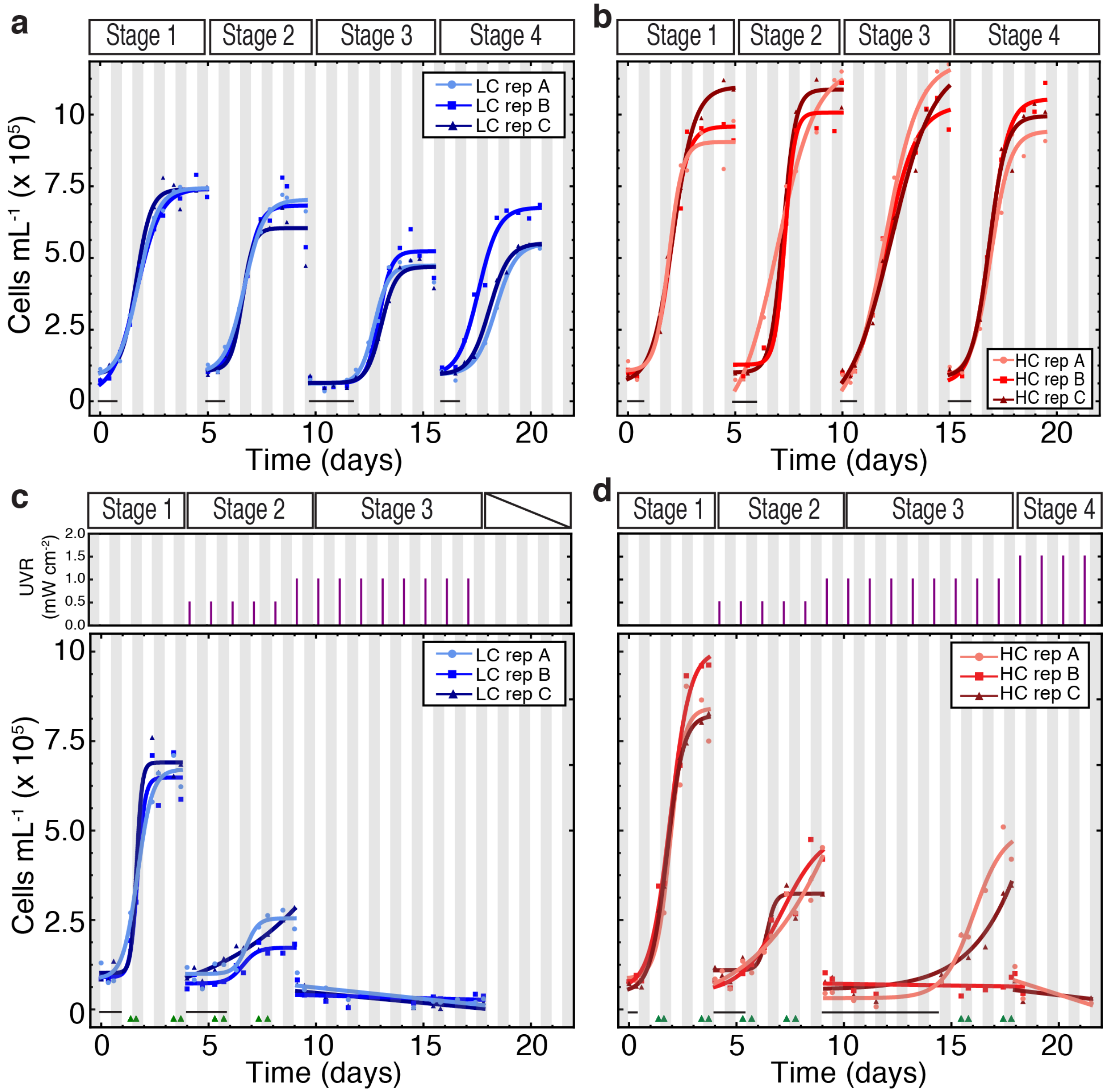

Supplementary Figure 1. Growth dynamics for individual LC and HC cultures during non-UVR conditions and the stress-test. *T. pseudonana* growth dynamics in mid-20th century LC conditions (300 ppm CO_2) (**a** and **c**) and projected HC conditions at the turn of the century (1,000 ppm CO_2) (**b** and **d**). During the stress-test (**c** and **d**), cultures received one hour exposures of an incrementally higher dose of UVR, starting with the second growth cycle or 'stage' (see Methods for detailed experiment design). White and grey bars indicate 12-hour light and 12-hour dark phases of the diurnal cycle, respectively. Purple bars in (**c**) and (**d**) indicate amount of UVR dose in mW cm^{-2}; cultures in (**a**) and (**b**) did not receive UVR. Black horizontal bars indicate length of recovery until re-initiation of growth. Green triangles mark time-points at which cells were harvested for transcriptomic analysis.

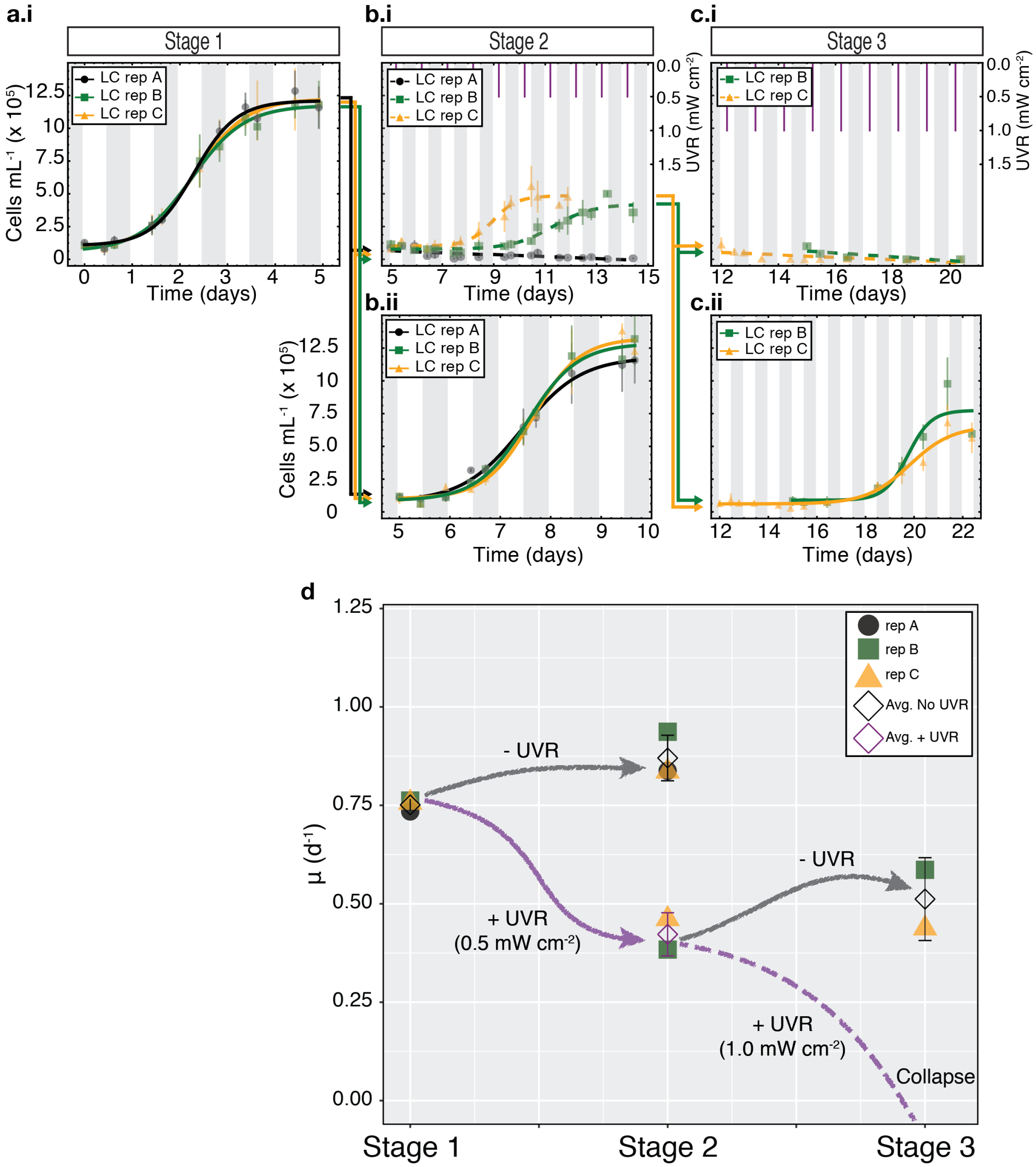

Supplemental Figure 2. **Isolating the role of UVR in collapse of *T. pseudonana* cultures**. Aliquots from triplicate cultures of *T. pseudonana* under LC conditions (300 ppm CO_2) in N-limiting conditions and 12:12 L:D cycles (**a.i**) were inoculated into two sets of triplicate reactors in stage 2, one set (**b.i.**) received 0.5 mW cm^{-2} UVR for 1-hour each day in the middle of the light cycle; the second set (**b.ii.**) experienced the same conditions as stage 1 cultures (i.e., no UVR). Aliquots from stage 2 cultures that received UVR treatment (**b.i**) were inoculated into two sets of reactors in stage 3; again one set received UVR (**c.i**) and the other did not (**c.ii.**). Vertical error bars denote the standard deviation of technical replicates (n = 4) of diatom cell counts for each time-point (**a-c**). Straight arrows indicate how cultures were split to inoculate a set of UVR exposed and non-UVR exposed cell cultures. The specific growth rates of replicate cultures from all three stages are plotted in panel (**d**), error bars represent the standard deviation of growth rates from each stage ($n_{\text{No-UVR-stage 1,2}} = 3$; $n_{\text{No-UVR-stage 3}} = 2$; $n_{\text{+UVR stage2}} = 2$).

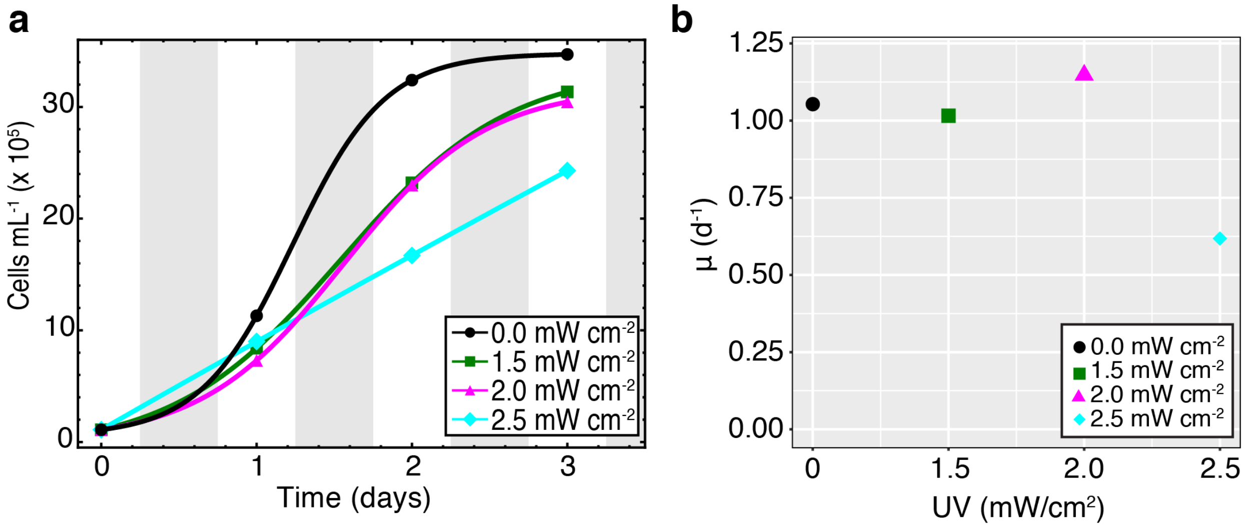

Supplementary Figure 3. Dosage response curves (a) at different UVR intensities at ambient CO_2 (~400 ppm) and their corresponding growth rates (b). Cells were grown in 1 L of non-limited (ESAW medium) bottle reactors under a 12:12 L:D regime and subjected to different doses of UVR for one hour in the middle of each light cycle.

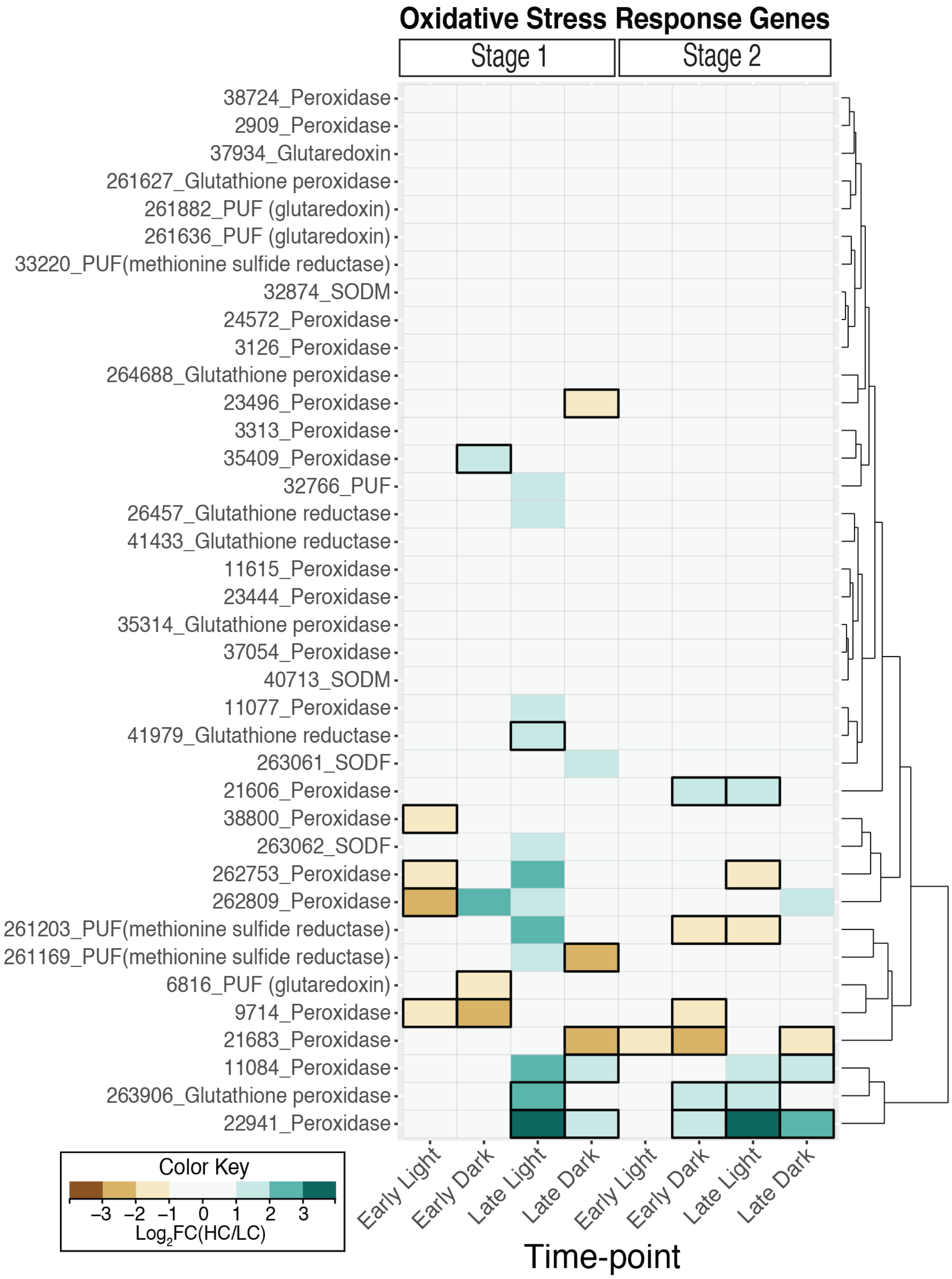

Supplementary Figure 4. Differential expression of oxidative stress response genes across stages 1 and 2 in HC and LC conditions. Expression analysis of oxidative stress response genes (n = 38) associated with increased production of reactive oxygen species (ROS), including peroxidases, glutaredoxins, and superoxide dismutases. Genes are grouped based on hierarchical clustering, and labeled with their transcript I.D. and putative function. Cells outlined in bold represent genes with a significant (p-value < 0.05) differential expression with a $\log_2$ fold change (HC/LC) ≥ 1 or ≤ -1. (SODM: superoxide dismutases, SODF: iron superoxide dismutases, PUF: protein of unknown function)

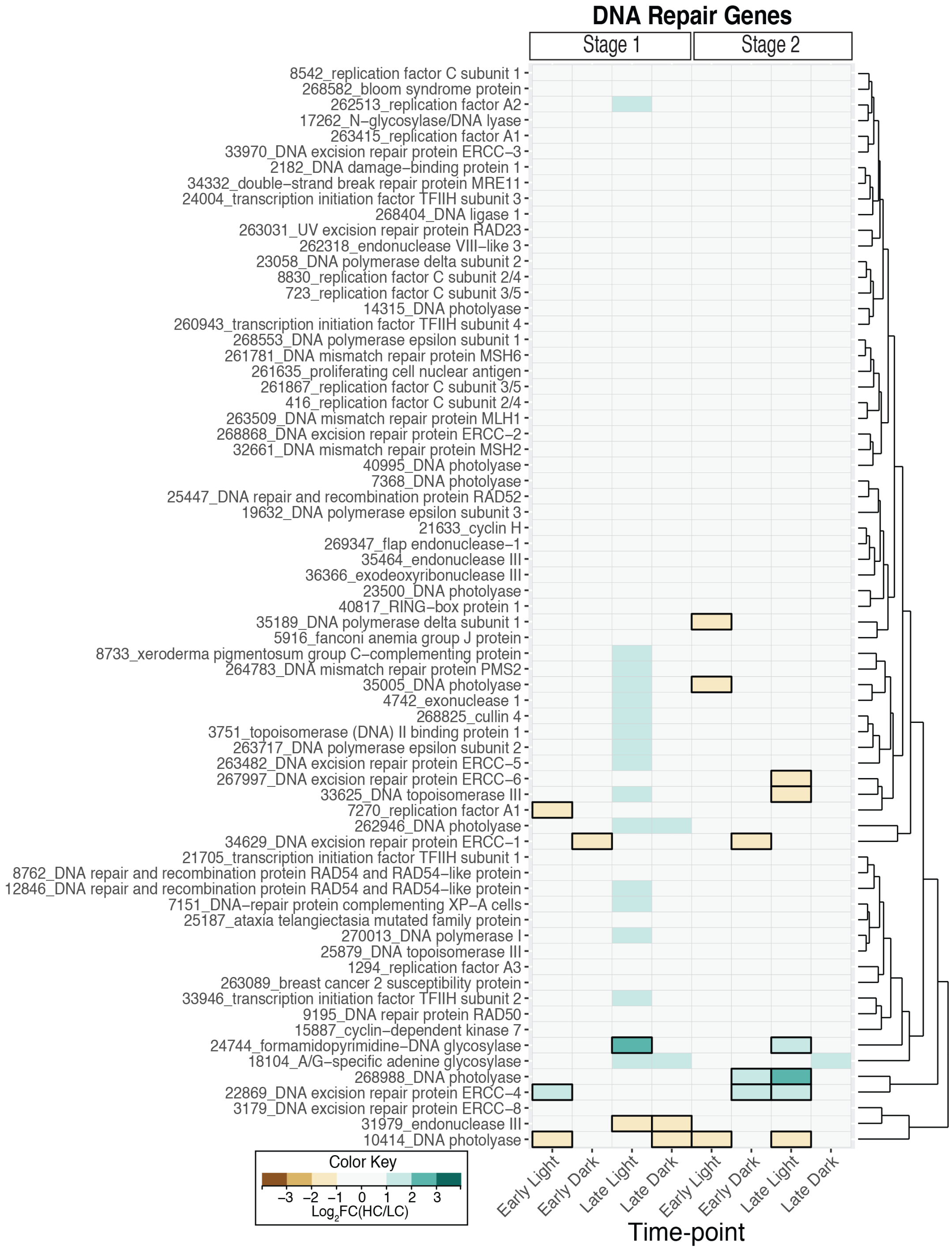

Supplementary Figure 5. Differential expression of DNA repair pathways across stages 1 and 2 in HC and LC conditions. When UVR damages DNA, repair mechanisms are expressed to limit corruption of the genetic code. Transcriptomic analysis of 69 DNA repair genes (i.e., homologous recombination, base excision repair, mismatch repair, nucleotide excision repairs, and DNA photolyases) revealed only subtle differences between carbon conditions. Genes are grouped based on hierarchical clustering of their gene expression. Cells outlined in bold represent genes with a significant (p-value < 0.05) differential expression with a $\log_2$ fold change (HC/LC) ≥ 1 or ≤ -1.

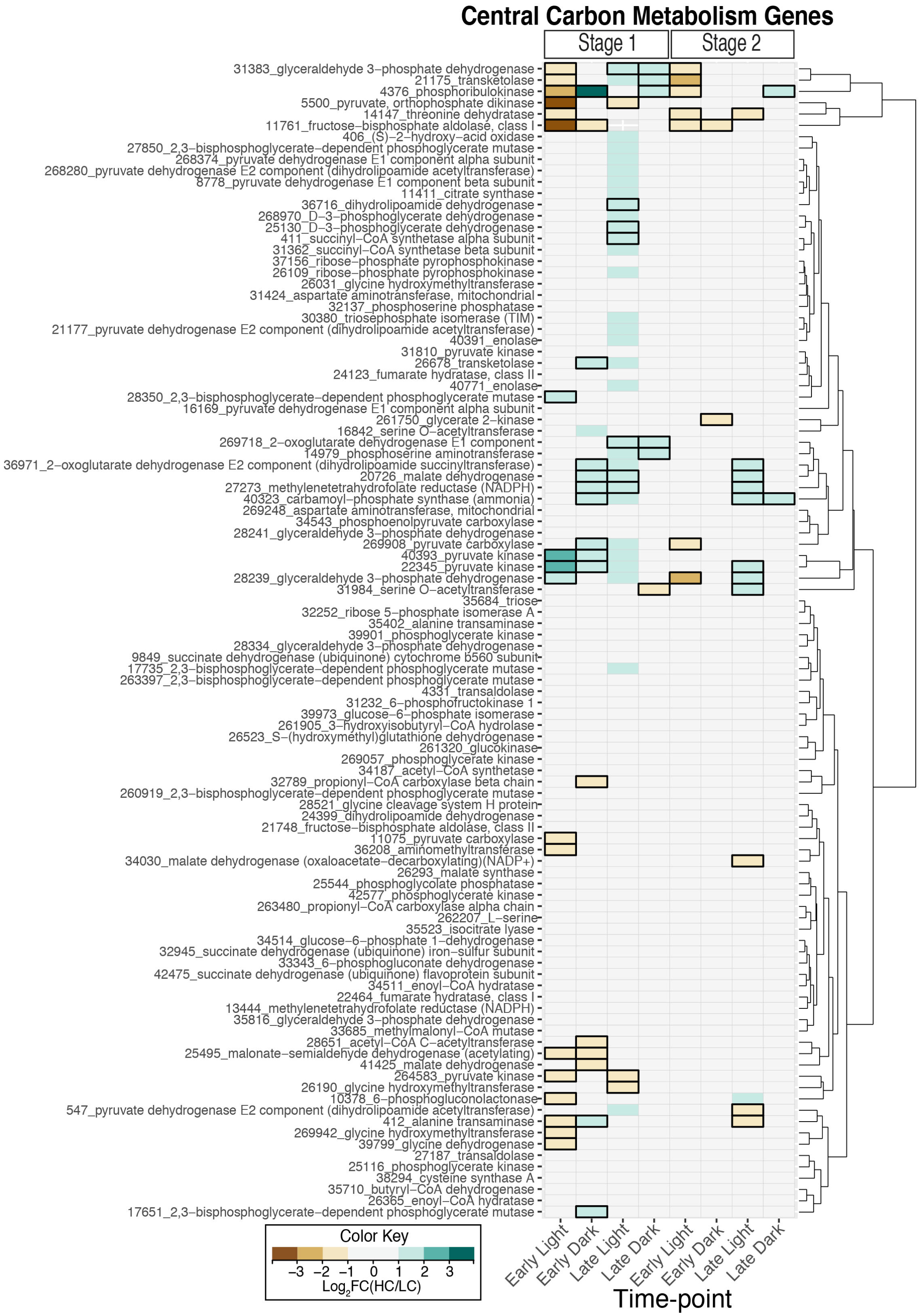

Supplementary Figure 6. Differential expression of central carbon metabolism genes. The differential expression from 102 genes consisting of the pentose phosphate pathway, glycolysis,

TCA cycle, and the Calvin cycle. Genes are grouped based on the hierarchical clustering of their differential expression. Cells outlined in bold represent genes with a significant (p-value < 0.05) differential expression with a $\log_2$ fold change (HC/LC) ≥ 1 or ≤ -1.

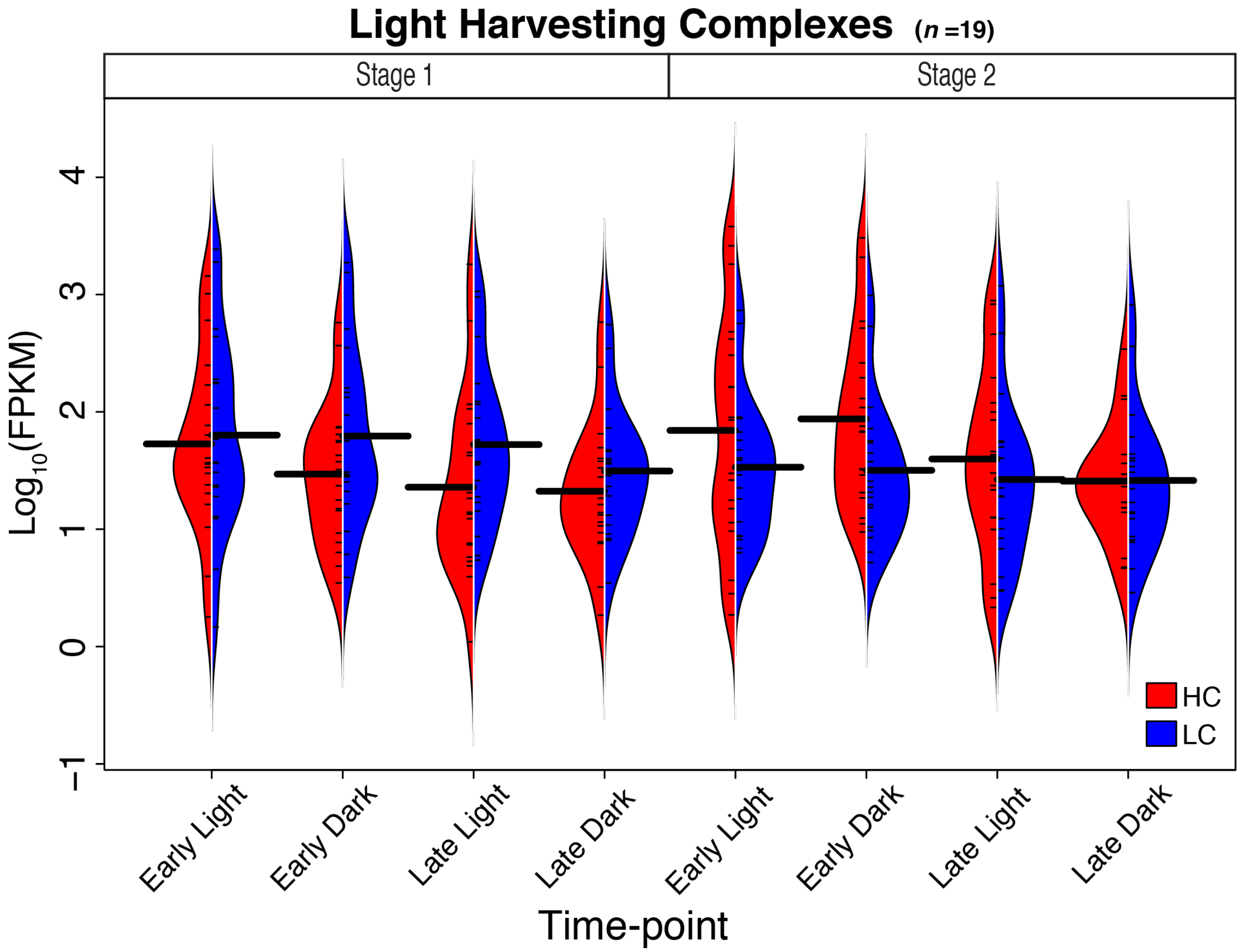

Supplementary Figure 7. Bean plots of 19 light harvesting complexes. There was no significant difference in expression of LHCs at any time-point (n = 19) between HC and LC conditions. Black bars indicate mean expression.

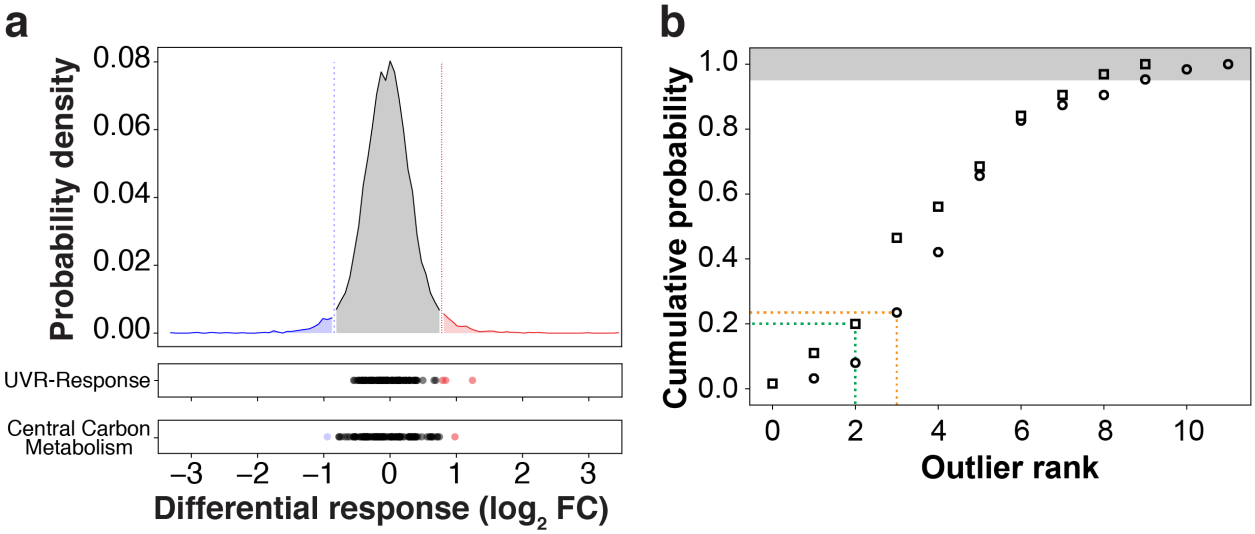

Supplementary Figure 8. UVR does not have a significant transcriptional effect on differential carbon response of UVR responsive and central carbon metabolism genes. (**a**) Distribution of the differential transcriptional response to carbon levels between stage 2 and stage 1 for a set of OSR and DNA damage responding genes (n = 107) and central carbon metabolism genes (n = 102). Three genes (colored dots) from UVR-response and two in the central carbon metabolism show a differential response, but are not significant (cross-validation; p-value = 0.765 and p-value = 0.8, respectively). (**b**) Probability distributions of the number of outliers computed from 10,000 random sets of 107 (circles) and 102 genes (squares). Orange and green dashed lines mark the observed outliers for the UVR response and central carbon metabolism gene sets, respectively. At least eight and nine outliers are required to indicate significance in sets of n = 102 and n = 107, respectively. Grey area indicates cumulative probability ≥ 0.95.

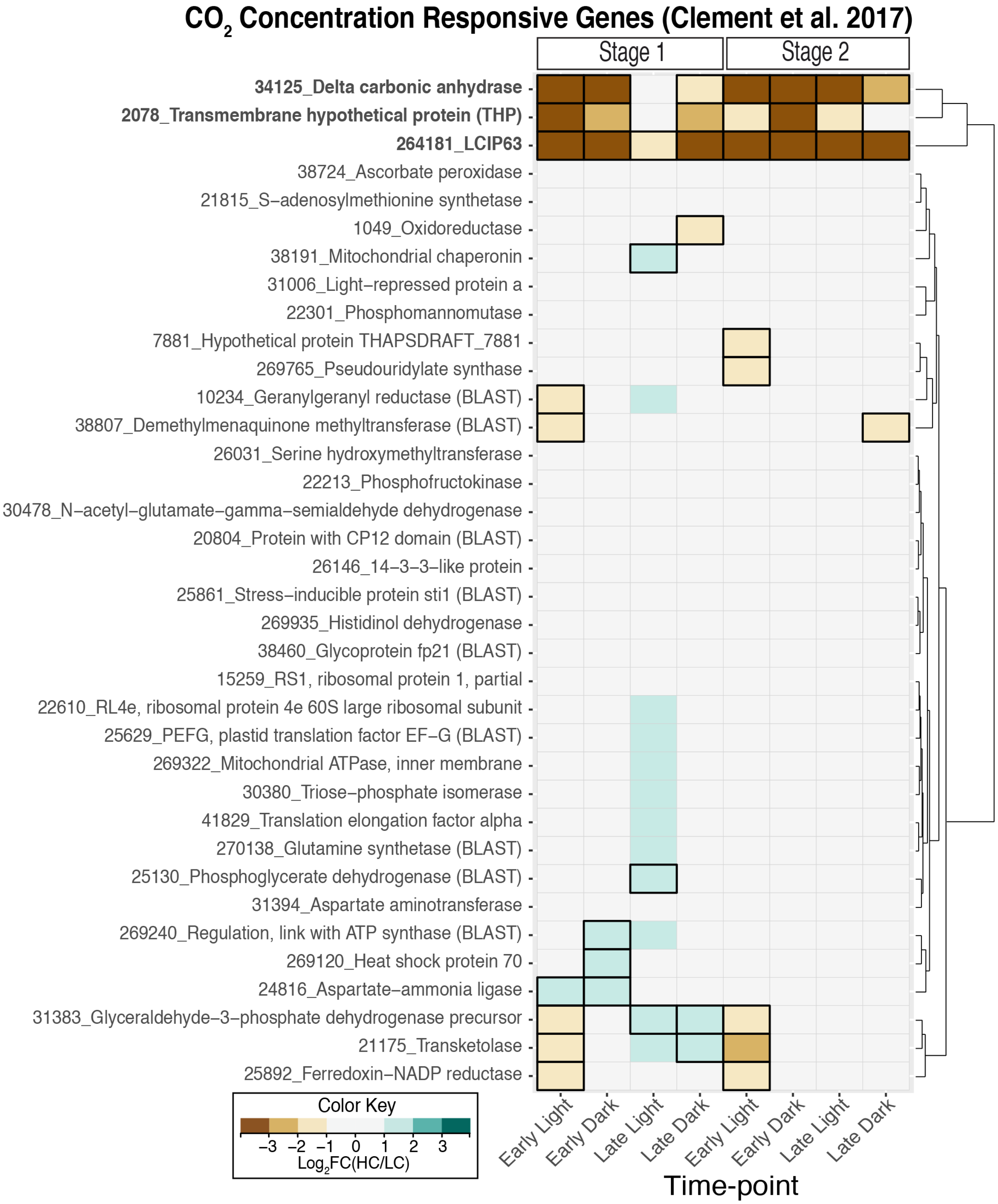

Supplementary Figure 9. Differential expression of 36 of 42 CO_2-responsive genes from Clement et al. 2017. Genes are grouped based on the hierarchical clustering of their gene differential expression. Cells outlined in bold represent genes with a significant (p-value < 0.05) differential expression with a $\log_2$ fold change (HC/LC) ≥ 1 or ≤ -1.

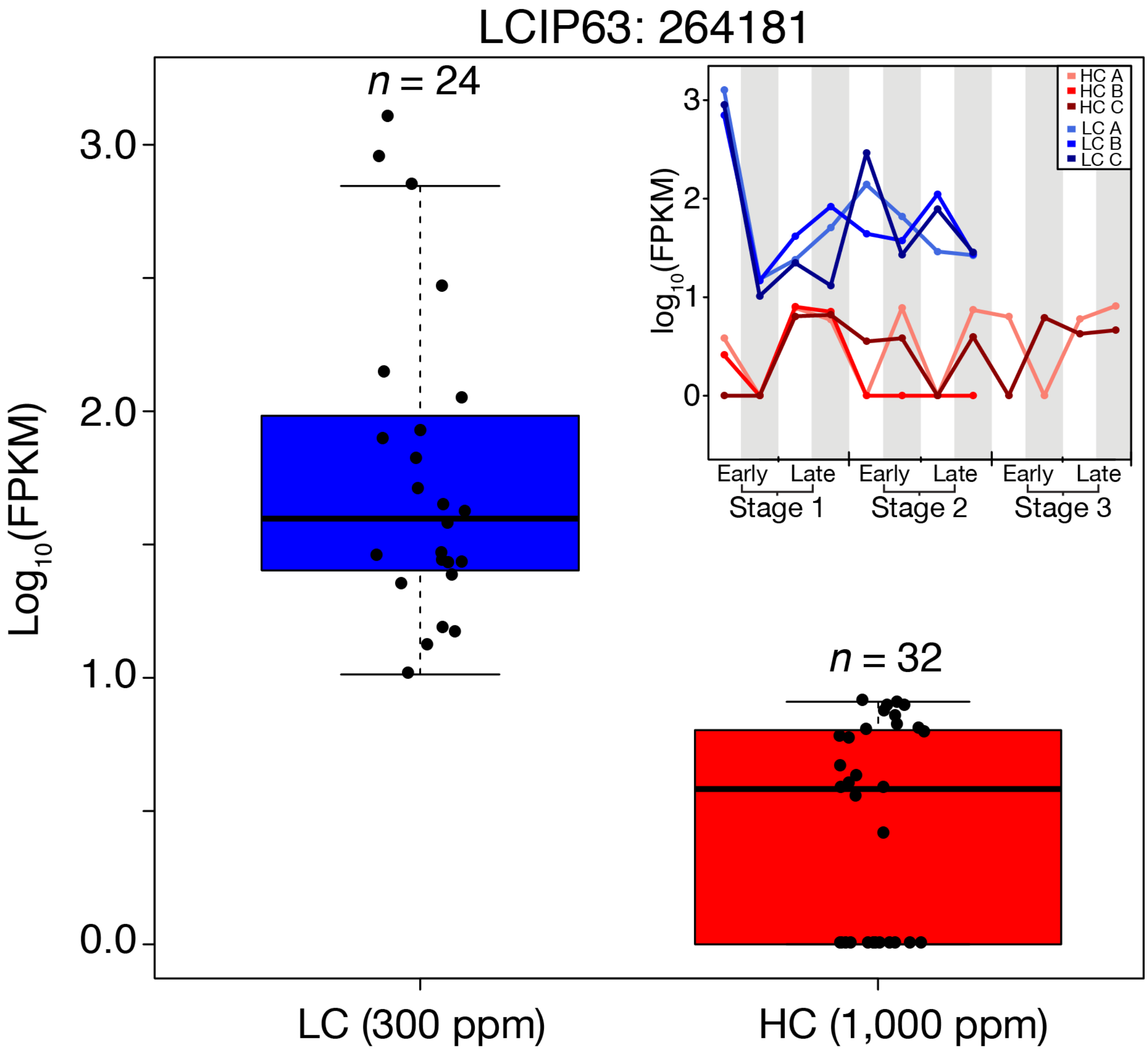

Supplementary Figure 10. Consistent over-expression of LCIP63 at low CO_2 during early or late phase of growth and in the light and dark cycles. Boxplots represent all transcripts throughout the stress-test, black center represents the median. The insert panel shows its regulatory pattern throughout growth and with UVR exposure.

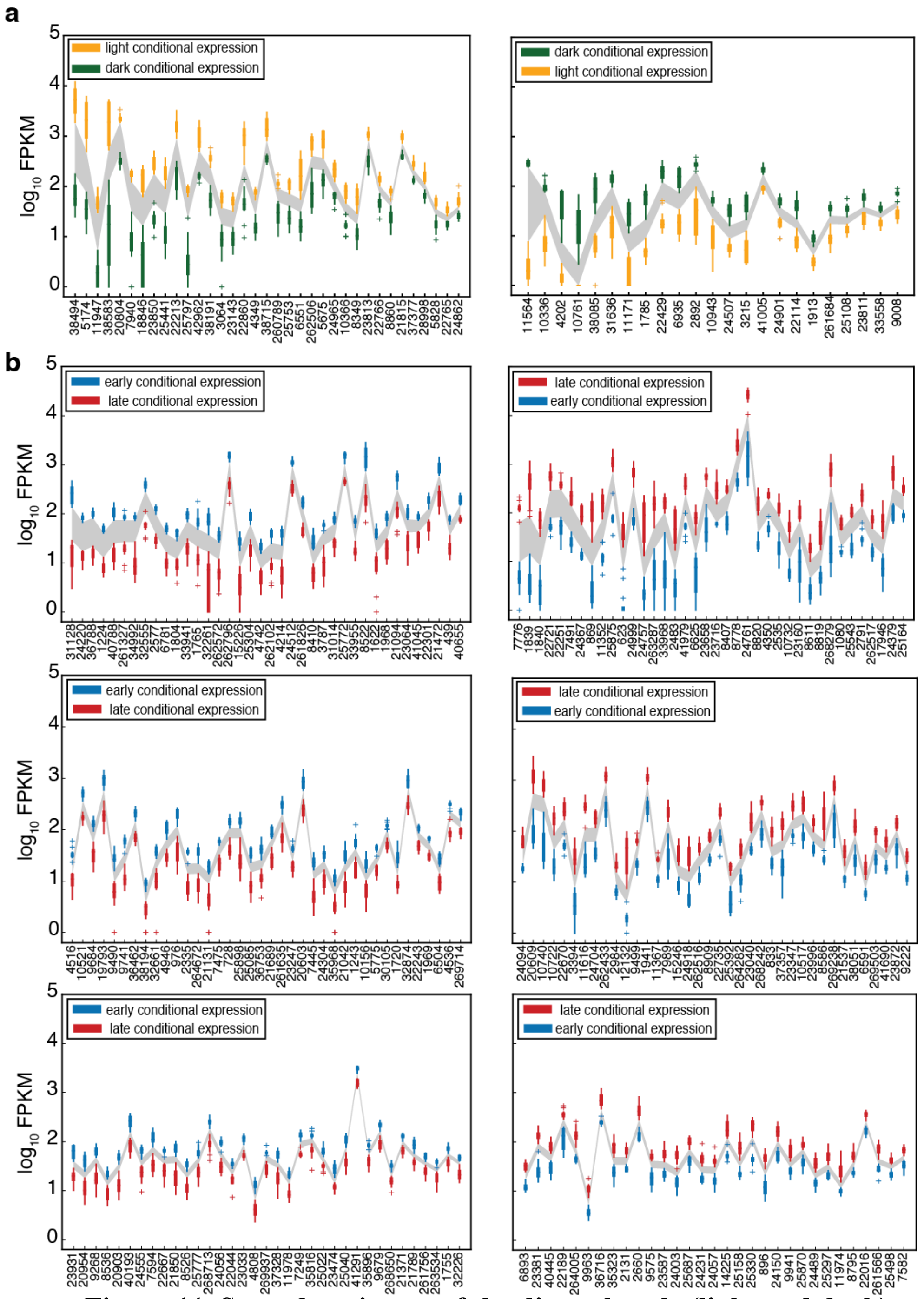

Supplementary Figure 11. State descriptors of the diurnal cycle (light and dark) and growth phase (early and late) of the first stage. Diurnal cycle descriptors (n = 58) were sub-divided into genes that had higher expression levels either during light (left panel) or dark (right panel**)** phase (**a**). Growth phase descriptors (n = 218) were also sub-divided into genes, which had higher expression either during early (left panels) or late (right panels) phase (**b**). Grey areas between boxplots represent non-distinctive expression. State descriptors are labeled with their transcript ID and a full list of the state descriptors including expression means is provided as Supplementary Data 1.

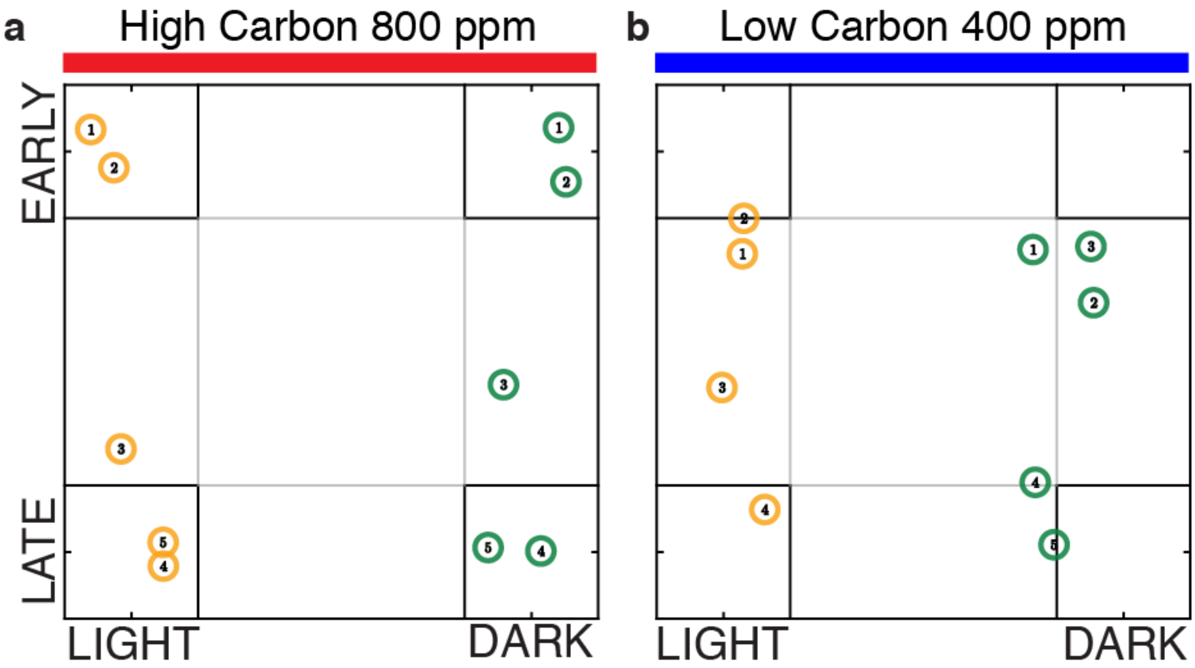

Supplementary Figure 12. Mapping transcriptomes from an independent study into 2D descriptor state space. State space generated using descriptors from this study were used to map transcriptome data sets from *T. pseudonana* batch cultures growing in 12:12 L:D cycles at 400 ppm (**a**) and 800 ppm (**b**)—see Ashworth et al. (2013). Circles (yellow and green) indicate transcriptomes from samples harvested during light and dark phase, respectively. Numbers within each circle represent the day the sampling occurred.

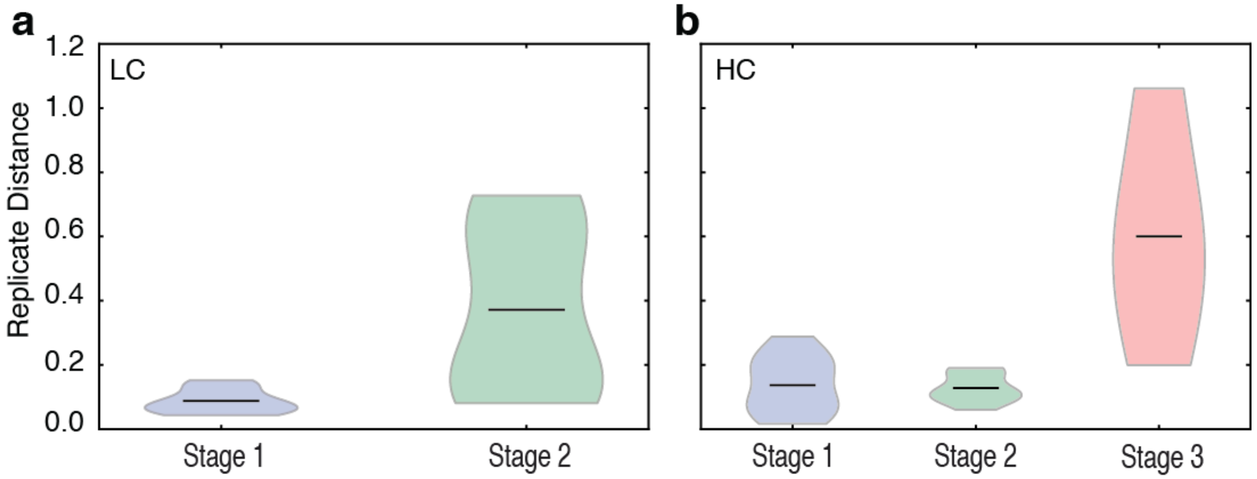

Supplementary Figure 13. Variability of culture replicates in state space prior to culture collapse. Euclidean distance between culture replicates at each sampling time-point within each stage in LC (**a**) and HC conditions (**b**). Increased variability was observed prior to collapse, in stage 2 for LC (Mann–Whitney U test; p-value = 0.000826) and stage 3 for HC conditions (Mann–Whitney U test; p-value = 0.00218).

Gene Annotation	Gene I.D.	LC (FPKM), *n* = 6	HC (FPKM), *n* = 6	$\log_2$ FC	p-value
(Tp_bZIP24a) regulator	Thaps10360	48.108	21.6088	-1.15466	0.0001
PRK08198	Thaps14147	104.299	37.4892	-1.47618	5.00E-05
hypothetical protein	Thaps2078	60.0438	7.90536	-2.92511	0.0001
**CdCA1**	**Thaps233**	**111.512**	**17.2395**	**-2.69341**	**5.00E-05**
**CdCA1**	**Thaps25840**	**922.25**	**168.591**	**-2.45163**	**5.00E-05**
COG2423	Thaps260953	506.649	127.067	-1.9954	5.00E-05
FMN_dh	Thaps3353	109.564	57.7226	-0.924568	0.00125
**CdCA1**	**Thaps34125**	**618.767**	**5.03526**	**-6.94119**	**5.00E-05**
PLN02389	Thaps34585	310.37	135.101	-1.19995	5.00E-05
Bestrophin superfamily	Thaps4819	156.696	13.8237	-3.50275	5.00E-05
Bestrophin superfamily	Thaps4820	180.514	34.7197	-2.37829	5.00E-05
hypothetical protein	Thaps6529	415.159	26.9684	-3.94432	5.00E-05
(Tp_bZIP24a) regulator	Thaps9903	95.3548	20.0291	-2.25121	5.00E-05

Supplementary Table 1. Significant down-regulation of CCMs and carbonic anhydrases (CA) in HC conditions. Samples at early growth phase during the light cycle for stages 1 and 2 had significant (p-value < 0.001) down-regulation of 13 of 21 transcripts from the previously identified CCM sub-cluster (Hennon et al. 2015) at HC compared to LC conditions. Highlighted in bold are 3 carbonic anhydrases part of the biophysical CCM pathway with significantly large down-regulation (> 5-fold). We used the computational tool cufflinks (Trapnell et al. 2012) for sample comparison of FPKM (Fragments Per Kilobase of transcript per Million mapped reads).

	Ashworth et al. 2013	Valenzuela et al.
Thalassiosira pseudonana	CCMP1335	CCMP1335
Diurnal cycle	12:12 Light:Dark	12:12 Light:Dark
Photosynthetically active radiation (PAR)	150 µmol photons $m^{-2} s^{-1}$	300 µmol photons $m^{-2} s^{-1}$
CO_2 conditions	400 ppm vs. 800 ppm	300 ppm vs. 1,000 ppm
Gene expression method	Microarray	RNA-sequencing
Limited nutrients	Nitrate, Phosphate, Silicate	Nitrate

Supplementary Table 2. Comparison of experimental parameters between two studies about ocean acidification effects on diatom transcriptomes. Summary table of the similarities and differences between Ashworth et al. (2013) and this current study.

Growing Conditions	Hypothesis	U Statistic	p-value	Significance
LC	Stage 1 vs. **Stage 2**	17.0	0.000826	yes
HC	Stage 1 vs. Stage 2	70.0	0.465	no
HC	Stage 1 vs. **Stage 3**	4.0	0.00902	yes
HC	Stage 2 vs. **Stage 3**	0.0	0.00218	yes

Supplementary Table 3. Culture replicate distances in state space increase prior to culture collapse. For LC, we observed increased Euclidean distance between culture replicates at each time-point within stage 2 compared to stage 1 (Mann–Whitney U test; p-value < 0.01). At HC conditions, we found no significant difference in replicate distance of time-points between stage 1 and 2, while distance increased significantly before collapse at stage 3 (Mann–Whitney U test; p-value < 0.01). Bold indicates stages prior to collapse.

Supplementary Methods

Batch culture acclimation and growth.

All experiments were performed with the model diatom *T. pseudonana* CCMP1335 (Provasoli-Guillard National Center for Culture of Marine Phytoplankton) were grown in custom 1.5 L photo-bioreactors with enriched artificial seawater (ESAW) medium modified to have reduced levels of nitrate (~65 μM). Cells were under a 12:12 h L:D diurnal regime with saturating light conditions at approximately 300 μmol photons m^{-2} s^{-1} (Phillips-F32T8 Lamps). Cultures were grown at 20 °C and would range to approximately 22 °C during the light cycle. To establish 300 ppm and 1,000 ppm CO_2 aeration conditions, two sets of thermal mass flow controllers (Aalborg GFC-17 (2 x air, 2 x CO_2) were used to proportion CO_2 scrubbed air (Drierite Model 106-C) and 100% CO_2. Mixed CO_2 concentrations were monitored with a Qubit Systems (Model S151) CO_2 analyzer. Photo-bioreactors were inoculated with axenic *T. pseudonana* and allowed to acclimate at 300 ppm and 1,000 ppm CO_2 for two consecutive growth cycles before being transferred to sterile nitrate limited photo-bioreactors. In order to characterize population recovery, inoculum was allowed to deplete nitrate by means of growth and transferred when the culture had a lowered photosynthetic efficiency (*Fv/Fm*) at or around 0.30. Cells were inoculated to approximately 1×10^5 cells mL^{-1} in triplicate for each set of CO_2 conditions (3 x LC, 3 x HC). During the first stage, no UVR was applied to the cultures. When cells depleted the media of nitrate and their photosynthetic efficiency had decreased between (0.30-0.20), an aliquot of cells from the previous stage was transferred to fresh sterile nitrate limited media initiating the next stage. During stage 2, cells at mid-day were exposed to a dose of UV-AB at 0.5 mW cm^{-2} (Daavlin 305-12BB UVB and 350 12 UVA lamps) for one hour. UV-AB exposure was monitored with a Sentry UV Light meter (ST-513). After each subsequent stage, the UVR dosage was increased by 0.5 mW cm^{-2}. To allow sufficient photosystem

recovery after exposure to UVR, sampling occurred during the light period, between 3-4 hours after UVR dosage, and dark period sampling occurred approximately 4 hours into the dark phase. Exponential and late exponential grown cells were harvested and vacuum filtered onto 0.2 μm filters (GTTP 47 mm, Millipore) for RNA extraction during the light and dark period (Fig. 1 triangles) and immediately flash frozen in liquid nitrogen. In order to isolate the effect of UVR on growth we performed an additional stress-test on LC cultures cells, however at each transfer event we inoculated into two sets of reactors, one set with UVR and the other without (Supplementary Fig. 2). For instance, at the end of stage 1, one set received the UVR treatment (0.5 mW cm^{-2}, Supplementary Fig. 2, b.i, dashed lines) while the other set did not receive UVR (Supplementary Fig. 2, b.ii, solid lines). Replicate cultures receiving UVR during stage 2 were then used to inoculate two sets of reactors at stage 3, again one set with UVR (1.0 mW cm^{-2}, c.i) and the other set without UVR (c.ii). All growth curves were plotted by fitting growth data to a logistic model. The equation is a function of time (*t*), with parameters A, μ, λ corresponding to maximum cell density (i.e., carrying capacity), growth rate, and lag phase, respectively (Kahm et al. 2010).

$$y(t) = \frac{A}{1+\exp\left(\frac{4\mu}{A}(\lambda - t)+2\right)} \qquad \text{(Supplementary Eq. 1)}$$

RNA extraction and library construction. Total RNA was extracted following the Spectrum Plant Total RNA kit (Sigma Aldrich-STRN50) protocol including on-column DNase digestion (Sigma Aldrich-DNase 10) as described by manufacturer. Total RNA samples were prepared using the Illumina Truseq Stranded mRNA HT library prep kit (cat# RS-122-2103). The samples were poly(A) purified and fragmented, resulting in indexed libraries of cDNA. Libraries were qPCR quantified on an ABI 7900HT real time PCR system (Applied BioSystems), using a KAPA universal library quantification kit (Kapa Biosystems- cat#KK484) for Illumina samples. DNA

1000 chips for the Agilent Bioanalyzer were used to check quality and size of cDNA samples. Samples were then normalized to 2 nM and a total of 5 µL per sample was pooled. The final pool was run on the Qubit Fluorometer (ThermoFisher Scientific) at a dilution factor of 200 using the dsDNA high sensitivity assay protocol. The pooled library was denatured and diluted according to the NextSeq 500 protocol. The library was run paired end on a high output 300 cycle v2 flowcell on the Illumina Nextseq 500 platform. Data was uploaded to Illumina BaseSpace.
