# Supplementary material for: Ocean acidification conditions increase resilience of marine diatoms
Source: Nat Commun. 2018 Jun 13;9:2328. doi: 10.1038/s41467-018-04742-3 (PMC5997998; doi:10.1038/s41467-018-04742-3)
Supplement: Supplementary file 3 — Description of Additional Supplementary Files [file 41467_2018_4742_MOESM3_ESM.pdf]

Description of Additional Supplementary Files:

Supplementary Data 1. A full list of state descriptors split into two spreadsheets, distinguishing the diurnal cycle (n = 58) and growth phase (n = 218). The lists of state descriptors include their median expression during the first stage and the weighted average of each descriptor that was used to compute the position score (see Methods). In addition to the gene I.D. of each descriptor has been provided with its gene name, UniprotKB/TrEMBL I.D., gene description, and gene ontology domain when applicable.
